# Sidelined during COVID-19: a narrative inquiry into how simulationists experienced the pandemic

**DOI:** 10.1186/s41077-021-00196-8

**Published:** 2022-01-10

**Authors:** Kim Leighton, Suzan Kardong-Edgren, Anna Jones, Gabriel Reedy

**Affiliations:** 1 Itqan Clinical Simulation & Innovation Center, Hamad Medical Corporation, PO Box 3050, Doha, 00000 Qatar; 2 Health Professions Education, MGH Institute of Health Professions, Charlestown Navy Yard, 36 1st Avenue, Boston, MA 02129 USA; 3 Faculty Development Lead, Module Lead for Masters in Clinical Education, School of Medical Education, King’s College London, Guy’s Campus, 1.26 Henriette Raphael Building, London, UK; 4 Reader in Clinical Education, Programme Director - Masters in Clinical Education, Faculty of Life Sciences and Medicine, King’s College London, Waterloo Bridge Wing G7, London, UK

**Keywords:** Healthcare simulation, Sidelined, Qualitative research, Narrative research, Simulationists, COVID-19

## Abstract

**Background:**

In the simulation community, colleagues who are no longer clinically practicing were often proximal to the COVID-19 response, not working in the frontlines of patient care. At the same time, their work as simulationists changed dramatically or was halted. This research explored the experiences of those simulationists who have clinical backgrounds but did not provide direct patient care during the initial pandemic response. The aim of this study was to allow those simulationists to share and have their stories heard.

**Methods:**

This qualitative research used a narrative approach to answer the research question: What were the experiences of those in the simulation community who did not contribute to the frontline patient care response during the early stages of the pandemic? A semi-structured questionnaire aimed at eliciting a story was disseminated through online simulation discussion boards. Data was collected through PHONIC with options to type or speak responses. Responses were analyzed using an inductive analytical process to identify themes or patterns in the narratives.

**Results:**

Thirty-six respondents completed the survey between August 1, 2020 and November 30, 2020. Narrative arcs were identified that illustrated the events, actions, thoughts and feelings representative of experiences shared by many simulationists. Two major themes emerged: Challenges and Opportunities. Challenges included feelings of guilt; frustration; overwhelmed, stressed and exhausted; being away from the action, being unused and underappreciated. Opportunities included leadership (evolution and innovation), personal development, and being a part of something.

**Conclusions:**

The findings reflect a snapshot in time of how simulation was viewed and used in the world during a pandemic through the personal stories of simulationists with clinical backgrounds who did not provide direct patient care. Sharing these narratives may inform future simulation development; however, it is vitally important that the emotions are recognized and acknowledged. Managers should ensure mental health resources and support are available to all staff, including those not deployed to the frontline.

“As a nurse, I wanted to put on scrubs and head to the Emergency Department (ED), even though it had been 20 years since I had last worked a clinical shift. But, deep down, I was so grateful that I didn’t have to work in the ED because we didn’t really know what COVID-19 was yet. These conflicting feelings made me feel very guilty.”

Throughout the COVID-19 [1] (coronavirus disease 2019) pandemic, and especially in the early stages, healthcare systems responded with great speed and at scale. As the pandemic progressed, healthcare workers were pressed into service, taking care of patients suffering from the novel viral infection, and its wide range of resultant health problems. Their stories and experiences have been documented and shared broadly through the media, in the academic press, and on social media. In the simulation community, there are a large number of colleagues who, for a variety of reasons, are no longer clinically practicing; these simulationists were often proximal to the response—being employed by hospital systems or working in clinical training programmes—but were not working directly in patient care roles. Their clinical identities, and their vast experience as clinicians, are invaluable for their work as simulation educators. Those same identities; however, have the potential to leave them feeling left on the sidelines, when their colleagues are engaging in patient care and they are not. For this study, sidelined was defined as a “point of view taken by a person who observes an activity or situation but cannot directly participate in it” (https://www.dictionary.com/browse/sideline). This research explored the experiences of those simulationists who have clinical backgrounds, but did not provide direct patient care during the initial pandemic response. In uncovering and articulating their stories, we show the depth of those clinical identities among simulationists (educators, administrators, managers, operations specialists, and researchers), and the breadth and nuance of their experiences of responding to the pandemic.

We began the study with an interest in the pandemic’s effect of the pandemic on simulationists with clinical backgrounds who were not providing clinical patient care. We found that although the study participants had roles other than front line clinical care, their experiences were varied; many experienced or created opportunities in the midst of the crisis. The term “sidelined” may be perceived as biased as it has connotations of being left out. The focus of our study was specifically on simulationists not in COVID-related clinical roles, and our initial hunch was that they may indeed have felt left out of the COVID response. However, as our findings make clear, many did report feeling away from the action, their experiences were diverse, and their contributions to their professions and community powerful. Any possible preconceptions we may have had were quickly overturned by the richness of the data. This study points to the strength of narrative in identifying a range of responses to a complex phenomenon.

The psychological, physical, and mental health of frontline workers is well studied in the literature. Those who are clinically trained and not able to participate in the response as they might normally in their clinical roles, are not well studied. Early guidance from public health experts was clear: the novel coronavirus represented such a threat that “…under rigid infection measures, non-essential personnel such as psychologists, psychiatrists, and mental health workers are discouraged from accessing isolation wards or isolation rooms designated for patients with COVID-19.” [2], p.76. This “emotional epidemiology” [3] of not being able to perform within the professional identity in which one is trained, is often neglected for those who are on the periphery of the pandemic frontlines. The psychological needs of frontline staff take precedence over those who are sidelined. In the current pandemic, non-essential health care personnel often included simulationists, psychologists, health care workers over 65, and clinicians who might be vulnerable, among others.

While not participating in frontline patient care, simulationists contributed where they could. In many places, they moved quickly to provide all of their hospital beds, heart and blood pressure monitors, intravenous (IV) pumps, and personal protective equipment (PPE) to the frontlines. This equipment sweep left them with essentially empty simulation centers. In some places, simulation centers were seen as a non-essential service and closed down. Those who were sidelined were often expected to shelter in place, further emphasizing a potential sense of isolation. The inability to work or return to work and the loss of one’s way of life are associated with depression [4]. Alternatively, some survivors in pandemics empowered themselves by being present for others, showing empathy, and providing psychological support for others [4].

Being sidelined can also cause a form of survivor’s guilt, or in the case of the pandemic, “shelter in place” guilt [5]. First described after the Holocaust, survivor’s guilt is a deep sense of guilt for doing too little to help others in danger. One intervention for the feeling of being sidelined is to seek support from and to provide social support to others [4]. The aim of this study was to allow those simulationists to share and have their stories heard.

## Methodology and method

This qualitative research used a narrative approach [6, 7] to answer the research question: What were the experiences of those in the simulation community who did not provide direct patient care during the early stages of the pandemic? The narrative approach sought to uncover and articulate aspects of the lived experience that may otherwise go unexplored, such as the experiences of clinicians who were not working in the frontline during the first phase of the COVID-19 pandemic. Narrative is a particularly powerful methodology to uncover the very personal nature of the lived experience, while providing a way of examining both the breadth and variety of those experiences. Furthermore, narrative helps to uncover how people make sense of their own complex, novel, and difficult situations. By seeking to understand this particularity of the experiences of health care professionals working away from frontline care during the pandemic, this paper argues that a nuanced understanding of the individual and the contextual can give us a richer view. As an approach, narrative seeks to describe events from the perspective of those who have experienced, and subsequently constructed a retelling of, that experience. Key elements to a narrative are the idea of change over time [8–10], transformation, and meaning-making. Narrative describes events, actions, experience, thoughts, and feelings in context. The teller of the narrative is both recounting these and attempting to make sense of them. Narrative is one of the primary ways that we make sense of experience [11, 12]. Narratives structure experience, making it more concrete, organized, and accessible. Moreover, narratives identify the significance of experiences, thoughts and feelings for the teller.

The prompts for this survey were constructed based on a narrative interview approach [13, 14] and as such the questionnaire was semi-structured and the questions aimed at eliciting a story [14]. Institutional Review Board approval was obtained prior to data collection. The survey was disseminated through the discussion boards of two large international simulation organizations, and furthered through social media posts on LinkedIn. Data was collected between August 1, 2020 and November 30, 2020 through Phonic.ai (San Franciso, CA) with options to type or speak the response. Verbal responses were transcribed and underwent sentiment analysis, using the artificial intelligence capabilities of Phonic.ai, and were categorized according to degree of positive, negative, and neutral statements within the response.

**Fig. 1:**
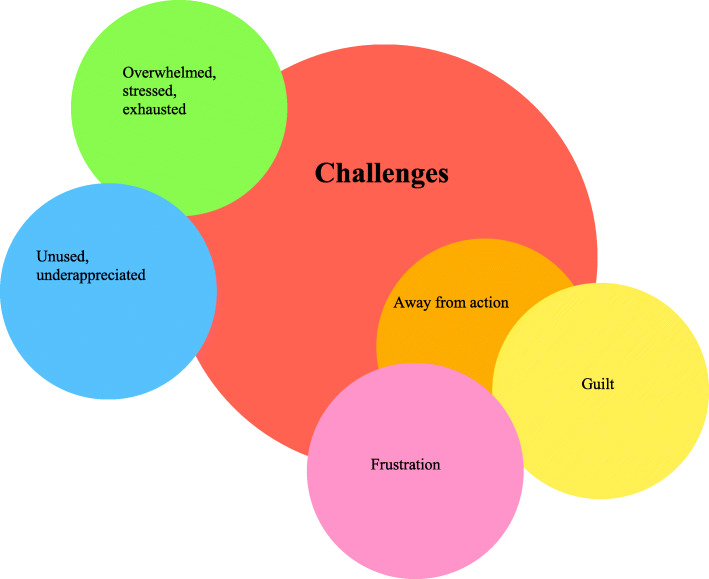
Challenges

**Fig. 2:**
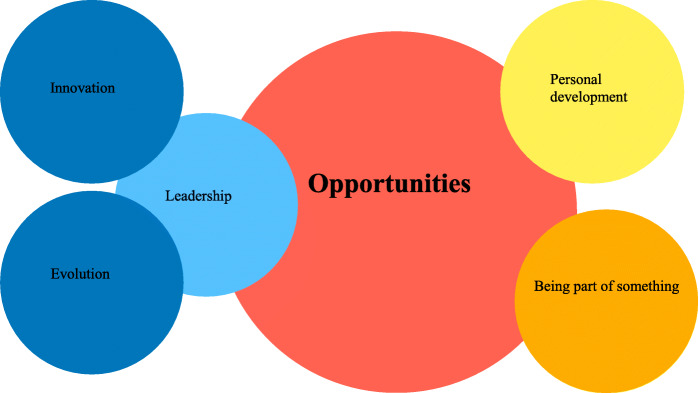
Opportunities

Responses to the survey questions were analyzed by the research team using an inductive analytical process, whereby the members of the research team engaged in a series of close readings of the narrative text responses and carefully considered the content and meaning of the responses. The analysis was designed to help the research team to identify themes or patterns in the narratives, and involved re-reading and validation through cross-checking across all transcripts [15]. Like Braun and Clarke, the research team embraced the analysis of the data as “reflect[ing] our view of qualitative research as creative, reflexive and subjective, with researcher subjectivity understood as a resource” [16]. The analysis process was iterative, productive, and time-consuming, as members of the research team challenged each others’ thinking and brought their own professional and personal subjectivity and interpretation to bear on the rich data provided by participants. The analysis process consisted of the following steps:

1. Each researcher read the transcripts in their entirety, assigning descriptive thematic categories (open coding).
2. The researchers shared their initial coding and agreed codes that would be used. This was an iterative process done several times with negotiation of differences in interpretation.
3. A final set of codes was agreed and each narrative recoded by all researchers (represented visually in Figs. 1 and 2).
4. Any discrepancies were discussed among the team and an agreement of interpretation was determined.
5. Researchers selected specific narratives to represent the overall narrative arcs or stories shared by participants.
6. Researchers selected extracts from the narratives to explicate and represent the themes present in the larger corpus of data.

The narratives presented in this paper were chosen because they told of a journey and had a clear narrative arc which showed changing perceptions and sought to explain the ways in which participants conceptualized their positions as non-frontline clinicians. The extracts are presented verbatim but hesitations and repetitions have been removed for ease of reading. Each narrative is from a different participant. These narratives are a small sample of the total number.

## Results

In this section, we detail the participants’ demographics, followed by a selection of overall narrative arcs. We then discuss the key themes interpreted from the data, with representative extracts from each theme.

### Participant demographics

Thirty-six respondents completed the survey, while a total of 51 answered at least one demographic question; all demographic data is reported in Table 1. Only those who responded to the open-ended item were included in the final analysis. Of the 36 responses, two-thirds were text and one-third were verbal. Of the verbal responses, sentiment analysis ranged from positive (*n* = 5) to negative (*n* = 4) with 2 neutral responses, and one unprocessed.

**Table 1:** Demographic findings

**Clinical background**		
Nursing	28	77.8%
Medical	3	8.3%
Dentistry	2	5.6%
Therapist (e.g., Physical, Respiratory, Occupational)	2	5.6%
Other Allied Health Professional	1	2.7%
Total	36	99.7%
**Simulation role (all that apply)**		
Education	29	43%
Administration	12	18%
Management	11	16%
Operations	11	16%
Research	5	7%
Total	68	100%
**Years as a clinician**		
< 10 years	5	14%
11–20 years	7	19%
21–30 years	13	36%
31–40 years	9	25%
> 40 years	2	6%
Total	36	100%
**Years in healthcare simulation**		
< 10 years	22	61%
11–20 years	11	31%
21–30 years	3	8%
Total	36	100%

### Narrative arcs: representative responses from participants

The research team began their analysis by considering the narrative arc or journey of the participants, in order to uncover the transformation and meaning making. Building on these, we then identified a set of themes that ran through the narratives. Both the narrative arcs and the themes illustrate the events, actions, thoughts, and feelings in a very particular context. While we do not suggest that either the narrative arcs or the themes from across the data are generalizable, we do strongly argue that they are representative of experiences shared by many simulationists who worked through the pandemic. It is through understanding the particularity of these stories that we can start to understand the broader experiences of those who worked through the pandemic.

Participant 49: Alberta, nursing

In 49’s narrative, they hold together both the positives and difficulties of the situation, capturing this tension. The story moves back and forth from the difficulties, outlining the seriousness of the situation, which is described as an “onslaught.” They talk of being “completely exhausted,” but then explain that the team has “come together and great things have happened.” Yet, while marking this achievement, they are still “yearning for a time when things will settle down,” and feels unsupported, sad and overwhelmed. They gain a lot of energy from reaching out to others and from participation, and that “action calms my anxiety.” So, participant 49 is oscillating through the range of experience, describing difficulties and sadness while at the same time acknowledging the ways in which, along with their team, they have risen to the challenges, found new ways and gained strength. The participant does not deny or underplay the challenges, but maintains the tension.

Participant 39: Idaho, nursing

Participant 39 expresses both the opportunities and stress of being away from the action. They begin their narrative by describing how they were rapidly pulled into finding new ways of providing education in a large health system and the challenges of developing new approaches. Yet they immediately follow this by expressing their concern for frontline workers who were struggling and the stress for people who could not be frontline. They described this stress as both emotional and moral, saying “we just wanted to be out there. We wanted to be on the frontlines.” Although they had not been a bedside nurse at their hospital, they also note that they were “ready and willing when the call came” and that at the time of recording they had been involved more directly in screening visitors and in flu vaccine clinics. Working virtually had enabled more connection with others, and while they had a useful and challenging role, they still felt the desire to be on the frontline.

Participant 41: Washington, nursing

Participant 41 speaks of the experience being at the same time exhausting and energizing. They describe the experience of having to develop new programmes very rapidly that met the changing needs of students who were unable to be in the clinical setting as frustrating but “that was also very exciting for me because I am an innovator. And so I really felt in my element, I felt empowered.” They described feeling very productive, even in the face of a rapidly changing situation, both in terms of the pandemic and the administrative directions. They describe working very hard, late at night but finding this rewarding: “it was incredibly stressful, as I said, but also incredibly exciting and energizing and rewarding…admittedly, I was, I think, drinking a lot of coffee and a lot of wine.”

Participant 27: New Hampshire, nursing

Participant 27 felt both guilt and relief at not being on the frontline. As a full time educator, they first described concern about being called to frontline clinical work and how to juggle that and full-time teaching. And as someone who was in a higher risk category, there was also relief at not being clinical: “so I guess a little part of me was relieved that I never had to do that or never be put in that position.” And as a caregiver for elderly parents, they had concerns about caring for and keeping them safe if they were in a more frontline role. Yet despite the concerns, they said “if I needed to, I would absolutely go out there and care from my community.”

These narratives show the participants holding different perspectives, often simultaneously; along with an awareness of the ambiguity of their explanations and an ability to hold this tension. Participants describe being at once stressed and energized; finding it difficult to be away from the action and yet seizing the opportunity to find new ways of working; and feeling both guilt and relief at not being on the frontline. Each narrative moves back and forth between the different perspectives, validating both. They do not seem to describe their perspectives as conflicting, but rather to tacitly acknowledge the complexities of the situation and their multiple responses.

### Themes identified in the responses

#### Challenges

The theme “challenges” was identified as respondents shared their experiences during the first months of the pandemic, including feelings such as *guilt* and *frustration*, being *overwhelmed*, *stressed*, *and exhausted*. They reported feeling a sense of responsibility to act, but were *not involved in the action*, resulting in *feeling unused and a lack of appreciation* for how simulation could help their peers and organization.

#### Guilt

Guilt was expressed for numerous reasons including lack of frontline involvement, worry about peers and newly trained clinicians, cancelation of standardized patients, but also because for various reasons, the respondents could not, or did not want to, be on the frontline.

“I was feeling slightly, I don’t know. Guilty, I guess, for not being a frontline worker and having sent out educated, trained so many young people that were now out there doing the frontline work.”

“I saw my fellow nurses on the frontlines; stressed, overworked, scared, and exhausted. . . many of us felt lost, helpless, and even guilty for not being on the frontlines.”

“I did not volunteer because I am at risk due to my age and felt badly about my decision.”

“For health reasons, I would have never been put on the frontlines anyway but this did not help the feelings of worthlessness: it really intensified the feeling. All-in-all, I know I did my part, but I often wonder if there was more I could have done or could be doing.”

However, some respondents verbalized resistance to going to the frontline by “refusing to go up there as I did not want to become a vector myself.” Another had just had major surgery and COVID-19 extended the respondent’s medical leave. “I had a longer recovery time and I was not complaining about that. I’m sorry. I’m sorry.”

#### Frustration

Frustration was often expressed as a lack of agency, as some respondents lost their jobs or remain furloughed, while others tried to get frontline positions but were denied.

“I was very frustrated not being on the frontline. I really wanted to get on a plane and fly to New York. But I knew I had my own family at home. Um, I had a job here that needed to be done.”

“So, even though my workload at least tripled, I felt helpless as a nurse/practitioner.”

“In my current work as an academic and simulationist, I feel useless.”

“While I feel the experiences I’ve been able to provide to students since COVID-19 have been valuable, I wish I could do more.”

#### Overwhelmed, stressed, and exhausted

Other respondents expressed their feelings of anxiety and stress related to early preparation for managing the environment, as well as how to work with their staff and family members to manage their anxiety. Lack of cohesion in responses from leaders and other departments negatively impacted respondents’ stress levels.

“Initially, it was very anxious. Um, a lot of anxiety about Covid (sic), about protecting the staff in making sure we got things right.”

“Stressed not knowing what I could do to support the nurses and other members of healthcare team.”

“And there was a large disconnect. I think, between the responsiveness of some of the, um shall I say, um, infection control guys plans for PP [personal protection] and staff expectations. And I found myself often in the middle of those.”

“But I also feel unsupported, and I feel sad and overwhelmed.”

Many respondents reported feeling exhausted and overwhelmed due to late nights working on new plans, responding to numerous problems, losing staff, and having no sense that relief was on the horizon. The situation in at least one simulation lab was reported as untenable due to the limited staff to meet expectations. One respondent noted that they were “more overwhelmed by dealing with a pandemic at large, and not so much overwhelmed by the work that we had to do to adjust.”

However, the majority were clearly overwhelmed by the work and new responsibilities, especially “work[ing] extra hard to make sure students and faculty were taken care of,” and “spending hours and hours in meetings developing activities to help our students.” As one respondent articulated it, they were “. . .putting out fires...all the time...every day. Relentlessly for 9 months and counting.” Another respondent characterized their response as “…exhausted and overwhelmed, and it has not let up. I see no foreseeable reduction…”

Despite the efforts described by the respondents, one reported they “felt uncertain if we were doing our best by the students. We really tried but wondered if it would be enough and were concerned about patient safety.” Several responded positively to the exhaustion they experienced, reporting feeling energized, rewarded, and having the realization that their work was important.

“I just felt truly as exhausting as it was, I felt truly energized to be able to help [others] come up with a workable plan that was successful and still helping our students meet the outcomes of their course.”

“. . . it felt is tiring as it was, it felt incredibly rewarding to collaborate with my colleagues to put out Resources immediately.”

“Now, I realized what I’m doing is very important and more important than my previous job; training future frontline workers to be safe, and helping to continue the supply of these needed workers.”

#### Away from the action

While most of the respondents experienced a great deal of stress and anxiety as they were immersed in the daily management of staff and environmental safety, policy creation, changing curriculum delivery, and ensuring the safety of their own families, there were several respondents who had no personal involvement in the clinical response, even if they sought it.

“I did try to get several jobs of trying to be on the frontlines, and I was unable to get there. Nobody was hiring. They were all in a hiring freeze. All the travel nursing around in the area. We’re not hiring at all.”

“I've offered to work clinically, however our local hospitals don't need the help. My university is not affiliated with a medical center, so there is no crossover work between academics and clinical practice. I wish my simulation expertise could be used to prepare local clinicians for COVID-19 procedures, such as protected intubation, but that opportunity is not available.”

“We actually haven’t had a single Covid (sic) patient in my department, so it was interesting. To, um, balance that line between getting people to be prepared and initially managing away the anxiety.”

#### Unused and underappreciated

Simulationists reported that they believed there were many opportunities for their personal expertise in simulation to have been used to create training that would have been beneficial for staff, clinicians, and ultimately the patients; however, their ideas and offers of assistance were not always met with enthusiasm. This left them feeling that their skills and knowledge were unused and that education was not appreciated, leading at least one participant to feel moral distress.

“I am hospital based and they have not embraced what simulation is as it compares to education and training. This frustrates me more than anything. COVID (sic) training was the opportune time to incorporate simulation experiences but it is not understood in my environment and now I am at the point of "learned helplessness!" There was such missed opportunity.”

“There were some times that it was difficult and, um, watching the stress of people, uh, that they were facing and not being able to go out there in sim because we knew they needed to practice. They needed to practice the new code blue changes. They needed to go practice this or that that was impacting them. But we couldn’t practice, and that was really hard, I think, emotionally and morally for a lot of us.”

“I wish my simulation expertise could be used to prepare local clinicians for COVID-19 procedures, such as protected intubation, but that opportunity is not available.”

“I don’t believe that that they fully understood what simulation could be capable of, even though they spouted platitudes about virtual and screen based training and all of that sort of stuff and being able to implement simulation with that.”

In other situations, participants reported that there were opportunities for simulation training; however, progress was not made or instructors took care of themselves without availing themselves of the assistance of the simulation teams. One respondent noted a lack of peers with the expertise to help move simulation forward during the crisis.

“All of the instructors kind of did some online things for themselves, and they didn’t necessarily need our help.”

“Some professors decided to abandoned there (sic) standardized patient simulations, only to turn around a month or two later and decided they wanted to conduct them. And some professors actually found themselves increasing standardized patient simulation.”

“Within my department… there is no one with expertise in education, let alone simulation, except me and I am not in any role to make a difference. I make subtle suggestions, use my debriefing skills to get leadership to have great ideas, but unfortunately, the infrastructure does not support true education and training of our healthcare workers.”

Even when simulation was enacted for training, it may not have been done at the same level that teams had been trained to perform at, leading to distress among the faculty, simulationists, and participants. In another example, the respondent felt simulation was only done to check off a box and not with the intent to ensure readiness to provide safe care.

“Unfortunately some of our sim faculty reverted to very medically oriented faculty led debriefing. This was very disappointing and when I spoke with one person who was actively oppressing IPE [interprofessional education] (physicians only discussions, the rest can take notes) discussions to ask about the debrief model they blew up at me and our relationship is not the same. Another reduced more than one participant to tears because they responded to the person's error with “you just killed the patient.” I almost did die on that hill but made my point and the person responded well to my debriefing. So much stress among people. Good people who want to help and are overwhelmed.”

#### Adaptation

Despite the many challenges shared by the respondents, there were many who shared how they adapted and what they learned from the challenges. Many of the adaptations and coping strategies were positive, although one respondent stated “Admittedly, I was, I think, drinking a lot of coffee and a lot of wine, so pick up in the beginning in the morning and at the end of the day.” Another found it “difficult as the days wore on to keep the glass half full as even some faculty expressed fear and chose to work remotely.” Others sought new opportunities to learn and network with the simulation community.

As another respondent put it, adaptation was something that they watched their students do, and realized they were doing it as well: “What I learned the most from this experience is that students adapt and you can learn other details of what students are learning when you change the process because you have to.”

### Opportunities

Other respondents who were sidelined from caring for patients on the front line, reported they were deeply involved in simulation during the pandemic and wanted to share their experiences. The theme “opportunities” was identified in some of the responses, with three subthemes including *leadership*, *being part of something*, and *personal development*. Leadership included both *innovation* and *evolution*.

#### Leadership-evolution

“…I think that faculty have released some of their sacred cows and really tapped into the creativity and are totally thinking differently about how we educate our students. They were forced to break away the chains to so many things that they held tightly to. For example, they are now doing video validations. Previously they thought that was a dumbest thing ever and then it would never, ever, ever, work. While I preached this over the years they now are believing more than ever in what we do.”

#### Leadership-innovation

Some participants worked in teams tasked with adapting simulation to the evolving COVID-19 environment. They were challenged and witnessed innovative work during the pandemic, and the use of simulation for training types, professions, and situations they had not previously considered. One respondent put it most clearly:

“What did I do during the pandemic shutdown? Simulation. Lots of simulation. Every procedure, policy and process related to COVID-19 in the health centre was created through and tested through simulation. Donning and doffing the various iterations of PPE, intubation in the ED, PICU [Pediatric Intensive Care Unit], NICU [Neonatal Intensive Care Unit], OR [Operating Room], the newly formed airway team and our flight team. code blue response, barrier shields or no barrier shields for intubation. Lots of aerosolized phosphorescent materials. Palliative care scenarios. Movement of patients through the health centre. Simulated admissions and emergencies in the new pandemic unit. Sim for families taking children with chronic illness home. Then there was making sim virtual or at least distanced. Deciding on priority education and priority learners that would be accommodated on site. Policies for the sim centre for use, entry, distancing, masking, cleaning - all rewritten and tested out in response to COVID-19. Videography and videodebriefing. writing scenarios.”

#### Personal development

Some participants were personally challenged and developed in new ways, seizing on the opportunity to develop new skills and explore new ways of working, supporting, and educating their colleagues and students.

“Wow. I found the experience challenging. I was given the task of creating a format for a 12 hour virtual clinical experience. I had to identify evidence to direct the development of the plan, create the plan, and train 15 full time and adjunct faculty on the plan-who then oriented their students on the process. I feel accomplished. The plan worked! I had the opportunity to share the plan with other faculty in different schools.”

“I was able to draw on all my expertise we had to go with exclusively virtual simulations with Zoom debriefings. This was not something I was used to doing. Using simulation to develop and refine the processes for dealing with any potential suspected or confirmed cases. We had to do some pretty quick adjusting.”

Other participants described personal development away from work.

“During my time off of work I have pursued many professional development opportunities and continuing education opportunities as well as completing some freelance work … [including] reviewing scenarios… as well as working with a team of subject matter experts to develop evidence-based immersive virtual reality end-of-program assessment scenarios.”

#### Being part of something

Several participants reported that they felt part of something that was making a difference during the pandemic. “All in all, as we were involved from day one and we had really a lot to do, it felt good - we were part of the global effort and felt very useful.”

“. . . particularly challenging and exciting time teaching the dental lab. Since March, it has been mostly synchronous seminars and some asynchronous. I had groups of students with professional nursing students assisting them carrying out 4-5 procedures in full PPE. Very rewarding- no time was wasted, these patients all turn up and students probably had more clinical learning in a straight 10-12 session with no breaks than ever before.”

“The main challenges were the necessity of developing training materials, especially the PPE ones, in many languages, as they applied not only to healthcare but also to supporting personnel (cleaning, janitors, etc). Delivering the trainings while maintaining hygiene and social distancing was also challenging. What we also did was put all of the respirators from our center (rest through a technical test and then give them to the clinical staff for usage on patients for as long as the crisis might take. All in all, as we were involved from day one and really a lot to do, it felt good - we were part of the global effort and felt very useful.”

## Discussion

These narratives provide a window into the participants’ reflections about their work in simulation during the unique experience of a worldwide pandemic. The experiences of our participants varied depending on administrative buy in, leadership, culture, and setting and yet despite their varied responses, some key themes emerged. We captured a unique set of data from simulationists willing to share their experiences during a time of great stress worldwide; their stories were personal, emotional, and reflective. These narratives highlighted the various dilemmas faced by respondents, such as wanting to be involved but unable to due to age, physical limitations, or family responsibilities. Others were sent home or laid off by systems that did not fully embrace or understand what simulation might have provided for the organization as far as training or preparation for caring for COVID-19 patients. This proved highly frustrating for some respondents who knew the power of simulation but were unable to persuade administration or leadership. Others were provided opportunities to demonstrate the power of simulation and worked to more than full capacity for weeks and months, training and preparing healthcare workers for COVID-19 patients, testing treatment modalitites, work flow, etc.

This study illustrates the complexities of healthcare simulation professionals’ responses to the pandemic. Their responses demonstrate the ways in which they structure and explain their experiences, in particular changes in situation, emotion or point of view [9, 10, 17]. Their reflections on their own situation—that of being “sidelined” from providing direct patient care—were multiple and sometimes conflicting, and yet they were able to articulate both the difficulties and the opportunities that this situation presented in a way that started to make some sense of a new and unexpected set of challenges. The participants articulated this tension—between frustration and achievement, guilt and relief, isolation and social connections, overwork and underemployment, innovation and backwards steps, pride and dismay, hope and fear.

The narratives illuminate the conundrums inherent in a situation that is unplanned, unexpected, and nearly universally stressful. Participants experienced sudden changes in their work situations and had little or no control over events. They responded with dismay, hard work, and for many, a high degree of adaptability. The narratives illustrate the ways in which people responded—through making online connections, developing new approaches (often at a rapid pace), and taking initiative. Yet alongside this, they expressed their frustration at the ways in which things were unfolding and their lack of agency. This study focused on clinicians who were not at the frontline and while many expressed disquiet at not being there to support colleagues and patients, others expressed a sense of quiet relief, due to heath concerns and caring responsibilities. While some were stymied in their simulation efforts, others explored and expanded the use of simulation in their organizations. The narrative arcs illustrate the ways in which the participants made sense of a profoundly challenging world and for many, holding and articulating their conflicting responses.

Narrative is a powerful tool for understanding clinicians’ experiences of the COVID-19 pandemic because experiences are complex, disjunctured, and overlayed with the profoundly emotional. Responses to the pandemic are both unique and mundane because each person’s experience is their own and yet the pandemic is now essential to the human experience. Listening to the stories of others allows us a view into the lives of others that resonates beyond each individual, reminding us of the fortuity of life.

## Conclusions

Qualitative research by its nature is not generalizable; however, this study does allow a snapshot in time of how simulation was viewed and used in the world during a unique period of history, as well as documentation of a sample of stories from clinically trained simulationists who were sidelined from providing direct patient care during the pandemic. These narratives present personal stories that might be recognizable to readers as they reflect on their own pandemic experiences.

The stark contrasts present in these narratives reflect the current understanding of simulation as an educational medium. While as simulationists, we hope that simulation is understood and embraced globally, we are always aware that this may not be so; these narratives suggest that much work remains to be done in sharing the power of simulation with others.

Importantly, and explicit in the reasoning of most qualitative work, the reflections and narratives about what was accomplished using simulation during the pandemic need to be widely shared, both inside the simulation community and beyond. The experiences reflected in the narratives from these participants are ones that resonated with members of the research team, and as such may be instructive to others who had very different experiences. Further, in uncovering these experiences, it is helpful to consider how simulation colleagues from different backgrounds and with different roles may have very different experiences of the same situation. Sharing these narratives—and indeed, personal experiences—with clinical colleagues, educators, operations staff, researchers, and administrators could potentially serve as a conversation trigger for future simulation development, prompting shared reflection and robust discussions about future directions and potential changes to current practices.

At the same time, it is vitally important that the emotions of our colleagues are recognized and acknowledged. We cannot overlook the fact that many of these colleagues also worked from home, away from their usual social networks, while also managing the safety of their families during a very stressful time. Managers need to ensure that mental health resources and other support mechanisms are readily available to all staff, including those who were not deployed to the frontline.

## Abbreviations

ED: Emergency department
COVID-19: Coronavirus disease 2019
IV: Intravenous
PPE: Personal protective equipment
PP: Personal protection
PICU: Pediatric intensive care unit
NICU: Neonatal intensive care unit
OR: Operating room

## Declarations

Ethics approval was obtained from the Research Governance Office of King’s College London (DPRF-19/20-14836). Consent was digitally provided prior to beginning the survey.
